# Diffuse osteosclerosis in a patient with systemic mastocytosis

**DOI:** 10.1210/jcemcr/luag228

**Published:** 2026-08-24

**Authors:** Huy H Do, Balasubramanian Krishnamurthy, Vivian Grill, Christopher Leow, Hanh H Nguyen

**Affiliations:** Department of Endocrinology and Diabetes, Western Health, St. Albans, Victoria 3021, Australia; Department of Endocrinology and Diabetes, Western Health, St. Albans, Victoria 3021, Australia; Department of Endocrinology and Diabetes, Western Health, St. Albans, Victoria 3021, Australia; Department of Medicine, The University of Melbourne, Parkville, Victoria 3010, Australia; Department of Haematology, Western Health, St. Albans, Victoria 3021, Australia; Department of Endocrinology and Diabetes, Western Health, St. Albans, Victoria 3021, Australia; Department of Medicine, The University of Melbourne, Parkville, Victoria 3010, Australia; Department of Medicine, School of Clinical Sciences, Monash University, Clayton, Victoria 3168, Australia

**Keywords:** mastocytosis, osteosclerosis, osteoporosis

## Abstract

Osteosclerosis refers to an abnormal increase in bone density resulting from excessive bone formation and/or reduced bone resorption. We present a case of a 31-year-old woman with acute-on-chronic abdominal pain, flushing, and diarrhea. Diffuse, mottled sclerosis involving the entire axial and appendicular skeleton was incidentally identified on imaging. Diagnostic investigations for osteosclerosis revealed a raised tryptase, leading to a bone marrow and trephine analysis. This demonstrated abnormally spindle-shaped mast cells, more than 15 in a group, with positive staining for tryptase which was consistent with systemic mastocytosis (SM). In the absence of “C” findings, a diagnosis of indolent SM complicated by diffuse osteosclerosis was made. Treatment with a histamine type-2 receptor antagonist provided mild symptomatic relief. In this report, we discuss the differential causes of osteosclerosis and the pathogenesis of bone manifestations related to SM, along with subtype-specific SM treatment options.

## Introduction

Compared to the increasing prevalence of osteoporosis, osteosclerosis is rare and often associated with serious underlying conditions. It refers to an abnormal increase in bone density resulting from disordered bone remodeling, usually involving both cortical and cancellous bone [1]. This can either result from an imbalance of mediators promoting osteoblastic activity, inhibiting osteoclastic activity or direct infiltration into bone tissue [2]. Some studies have used elevated Z-scores as a surrogate marker of high bone density; however, osteosclerosis remains a radiologic diagnosis rather than defined by dual-energy x-ray absorptiometry (DXA) [3]. Importantly, increased bone density does not equate to preserved bone strength, and affected individuals may remain at risk of fragility fractures [1, 4].

Osteosclerosis is frequently secondary to one of several diseases, some of which carry a poor prognosis. These include hematological malignancies, metastatic malignancies, renal osteodystrophy, and infiltrative disorders, such as systemic mastocytosis (SM). Recognition of this radiological finding should therefore prompt a thorough investigation to identify reversible or treatable causes. By reporting this case of diffuse osteosclerosis in a young female individual, we aim to contribute to the limited literature on osteosclerosis and highlight the current challenges managing SM-related bone disorders.

## Case presentation

A 31-year-old woman presented with acute-on-chronic abdominal pain, flushing, and watery diarrhea. She had 8 years of diarrheal symptoms which had worsened in the week prior to her presentation. She denied any significant weight loss. Her medical history was significant for polyendocrine metabolic ovarian syndrome, bipolar affective disorder (on lithium), and hidradenitis suppurativa.

Physical examination revealed a generally tender abdomen, no skin rashes, organomegaly or lymphadenopathy. Initial biochemistry revealed significantly raised inflammatory markers, with a total white cell count of 32.6 × 10^3^/μL (SI: 32.6 × 10^9^/L) (reference range, 4-11 × 10^3^/μL [SI: 4-11 × 10^9^/L]) comprising mainly neutrophilia [23.8 × 10^3^/μL (SI: 23.8 × 10^9^/L) (reference range, 2.0-8.0 × 10^3^/μL) [SI: 2.0-8.0 × 10^9^/L], and raised C-reactive protein (CRP) 202 mg/L (SI: 1923 nmol/L) (reference range, 0-10 mg/L [SI: 0-95 nmol/L]). Her hemoglobin was 14.9 g/dL (SI: 149 g/L) (reference range, 11.5-16.5 g/dL [SI: 115-165 g/L]) and platelet count of 273 × 10^3^/μL (SI: 273 × 10^9^/L) (reference range, 150-450 × 10^3^/μL [SI: 150-450 × 10^9^/L]). She had normal renal function with creatinine 0.88 mg/dL (SI: 78 μmol/L) (reference range, 0.45-1.00 mg/dL [SI: 40-90 μmol/L]) and mildly deranged liver function tests (bilirubin 0.94 mg/dL [SI: 16 μmol/L] reference range, 0-1.17 mg/dL [SI: 0-20 μmol/L]); alanine aminotransferase [ALT] 57 U/L [SI: 950 nkat/L] reference range, 0-30 U/L [SI: 0-500 nkat/L]; aspartate aminotransferase [AST] 46 U/L [SI: 766 nkat/L] reference range, 0-30 U/L [SI: 0-500 nkat/L]; alkaline phosphatase [ALP] 117 U/L [SI: 1950 nkat/L] reference range, 30-110 U/L [SI: 500-1833 nkat/L]; and gamma-glutamyl transferase [GGT] 158 U/L [SI: 2633 nkat/L] reference range, 0-30 U/L [SI: 0-500 nkat/L]) but without evidence of synthetic dysfunction (albumin 4.2 g/dL [SI 42 g/L] reference range, 3.6-4.9 g/dL [SI: 36-49 g/L]).

Stool and blood cultures were unremarkable, and a computed tomography (CT) scan of her abdomen did not demonstrate radiological evidence of colitis. Instead, it revealed diffuse, mottled bone sclerosis. This was found to involve the entirety of her axial and appendicular skeleton. There was no evidence of hepatosplenomegaly. No inguinal, pelvic, retroperitoneal, or mesenteric lymphadenopathy was appreciable. There were nodular, consolidative changes at the right lower lobe of her lung which appeared to be infective. She was discharged with a presumed diagnosis of community-acquired pneumonia and referred to the endocrinology department for outpatient workup of osteosclerosis.

## Diagnostic assessment

**Figure 1:**
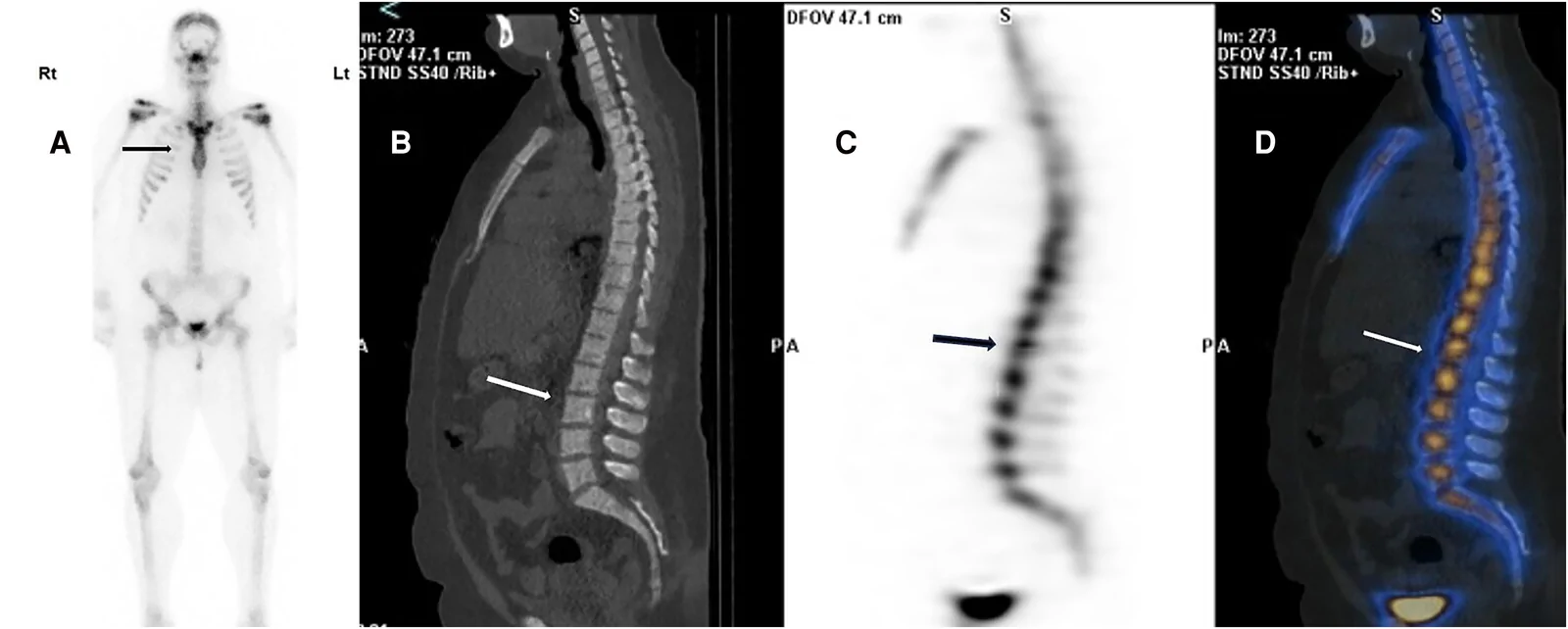
Panel A: neck-tie sign on bone scintigraphy. Panel B-D: diffuse crisp radiotracer uptake throughout the spine on a nuclear medicine whole body bone single-photon emission CT (SPECT)/CT scan.

A subsequent nuclear medicine whole body bone single-photon emission CT (SPECT)/CT scan demonstrated the neck-tie sign of the sternum (Fig. 1) and diffuse crisp radiotracer uptake throughout the rest of the skeleton consistent with a superscan pattern, an image appearance representing intense osteoblastic activity (Fig. 1) [5]. The neck-tie sign is thought to represent expansion of the manubrium and sternal marrow without concurrent expansion of the manubriosternal joint, with increased tracer uptake on bone scintigraphy giving an appearance of a neck-tie [6].

A bone mineral density (BMD) scan revealed a significantly elevated Z-score of 2.9 of the left total hip. At the lumbar spine and left femoral neck, the BMD Z-scores were 1.7 and 1.8, respectively. A bone formation marker (procollagen type 1 N-terminal propeptide 95 μg/L [reference range, 15-70 μg/L]) was elevated, while a bone resorption marker (C-terminal telopeptide 480 ng/L [reference range, 150-800 ng/L]) was normal.

Investigations for causes of diffuse osteosclerosis ruled out malignancy (normal electrophoresis, flow cytometry on peripheral blood, and mammography). Urine fluoride was not elevated at 1.2 mg/L (SI: 63 μmol/L) (reference range, <2.0 mg/L [SI: <105 μmol/L]). Other investigations included a normal corrected serum calcium 9.9 mg/dL (SI: 2.46 mmol/L) (reference range, 8.6-10.3 mg/dL [SI: 2.15-2.65 mmol/L]) paired with a normal parathyroid hormone (PTH) of 55.6 pg/mL (SI: 5.9 pmol/L), (reference range, 18.8-81.1 pg/mL [SI: 2.0-8.5 pmol/L]), and vitamin D was 20.43 ng/mL (SI: 51 nmol/L) (reference range, >20 ng/mL; SI >50 nmol/L). Hepatitis C serology was negative in addition to a lower CRP 21 mg/L (SI: 200 nmol/L) and neutrophil count had normalized (8.7 × 10^3^/μL [SI: 8.7 × 10^9^/L]). Thyroid function tests demonstrated subclinical hypothyroidism not requiring treatment (thyroid-stimulating hormone 8.29 mIU/L, [SI: 8.29 μIU/mL] (reference range, 0.4-4.2 mIU/L; SI: 0.4-4.2 μIU/mL), with free thyroxine (T4) 0.88 ng/dL (SI 11.3 pmol/L) (reference range, 0.8-1.8 ng/dL; SI: 10.0-23.0 pmol/L).

**Figure 2:**
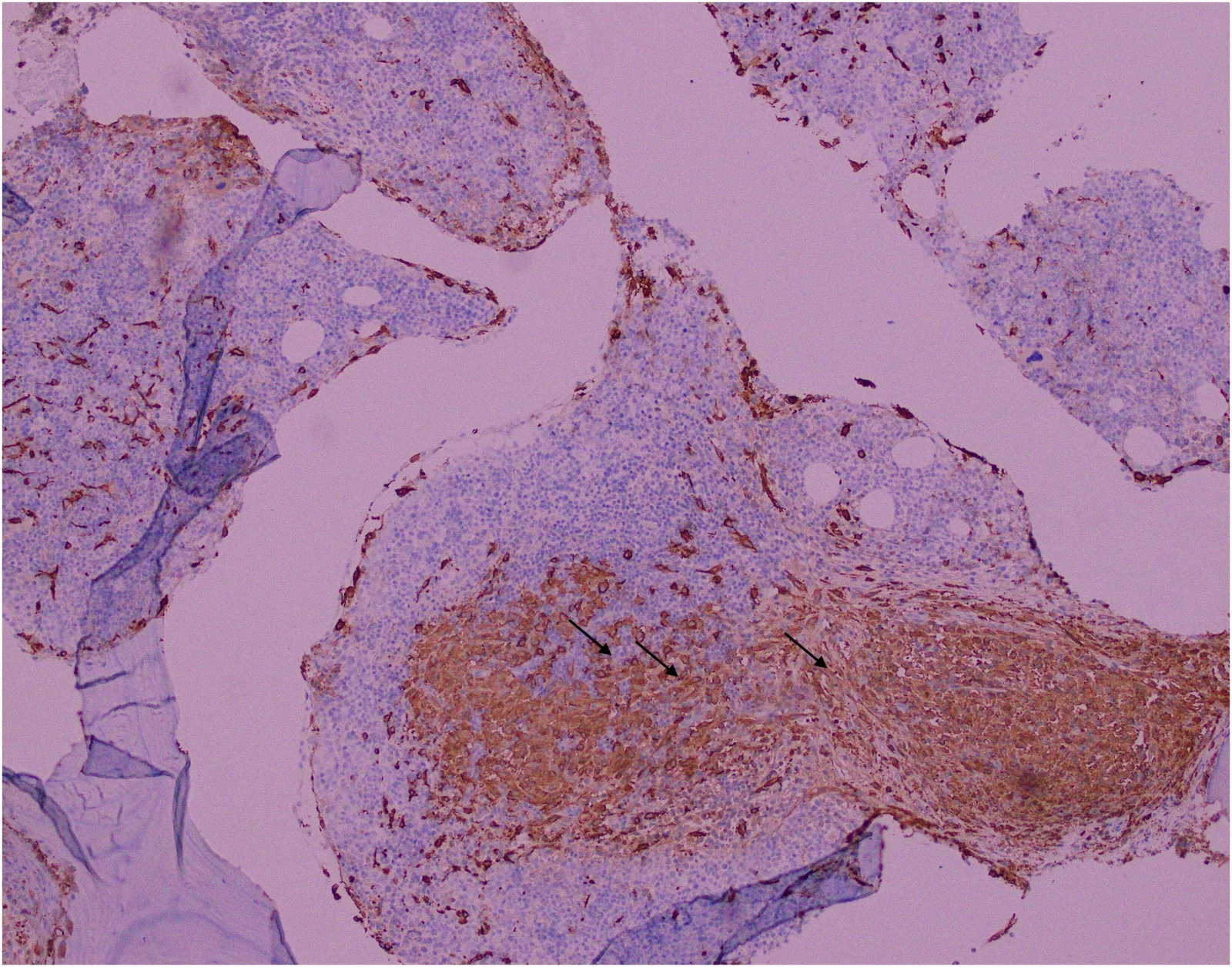
Trephine sample demonstrating spindle-shaped mast cells, with positive staining for tryptase. Microscope magnification 100×.

Further investigation revealed an elevated tryptase level of 126 ng/mL (SI: 126 μg/L) (reference range <11.4 ng/mL, SI: <11.4 μg/L). Bone marrow biopsy yielded a “dry tap” aspirate with trephine demonstrating abnormal aggregates of spindle-shaped mast cells >15 in a group, with positive staining for tryptase and CD25 (Fig. 2). No other hematological malignancy was identified. Peripheral blood *c-KIT D816 V* testing and all-heme next-generation sequencing (to identify other somatic mutations which may signify a poor prognosis) yielded no somatic variants.

A diagnosis of diffuse osteosclerosis secondary to SM was made. In the absence of “B and C” findings, her disease was classified as indolent SM (Table 1).

**Table 1:** WHO diagnostic criteria for systemic mastocytosis (SM) (referenced from Pardanani [7])

Major criterion
Multifocal dense infiltrates of mast cells (≥15 mast cells in aggregates) in bone marrow biopsies and/or in sections of other extracutaneous organ(s)
**Minor criteria**
≥25% of all mast cells are atypical cells (type I or type II) on bone marrow smears or are spindle-shaped in mast cell infiltrates detected in sections of bone marrow or other extracutaneous organs
KIT-activating KIT point mutation(s) at codon 816 or in other critical regions of KIT in bone marrow or another extracutaneous organ
Mast cells in bone marrow, blood, or another extracutaneous organ express one or more of: CD2 and/or CD25 and/or CD30
Baseline serum tryptase concentration > 20 ng/mL (in the case of an unrelated myeloid neoplasm, an elevated tryptase does not count as an SM criterion. In the case of a known HαT, the tryptase level should be adjusted
**The diagnosis is SM if at least 1 major and 1 minor or 3 minor criteria are fulfilled**

## Treatment

Treatment was aimed at providing symptomatic relief from degranulation, with a histamine type-2 receptor antagonist (ranitidine 300 mg daily) initially used, and she subsequently trialed a maximum-dose proton pump inhibitor (pantoprazole 40 mg daily), resulting in improved gastrointestinal symptoms.

## Outcome and follow-up

Currently, this patient's diffuse osteosclerosis has remained stable on serial CT imaging over 14 months, and she remains asymptomatic in relation to her bone disease. She is currently under regular hematology outpatient surveillance for development of “B” or “C” symptoms, which may require systemic cytoreductive therapy.

## Discussion

We present a case of diffuse osteosclerosis secondary to SM. Although rare, osteosclerosis has significant clinical implications, as it is frequently secondary to several serious conditions (Table 2), including malignant (infiltrative lymphoma or leukemia, osteoblastic metastases) or hematological (myelosclerosis, mastocytosis) disorders [8]. Other causes include congenital (osteopetrosis) and metabolic (hypoparathyroidism) processes.

**Table 2:** Differential diagnoses for osteosclerosis (adapted from Al Salam et al. [8] and Unger et al [9])

Differential diagnoses	Relevant investigations
**Hematological disorders**	
Myelofibrosis	Blood film Bone marrow and trephine (BMAT) Protein electrophoresis Free light chain ratio
Sickle cell disease	
Osteosclerosing multiple myeloma	
**Mastocytosis**	Serum tryptase, BMAT
**Malignancy—osteoblastic metastases**	
Prostate and breast	Breast mammography Renal tract imaging
Lymphoma: infiltrative	Flow cytometry Blood film Biopsy
Leukemia: infiltrative	
**Metabolic**	
Renal osteodystrophy	UEC, PTH, CMP, BTM, bone biopsy
Hypothyroidism	TFT
Hypoparathyroidism	PTH, CMP
**Poisoning**	
Fluorosis	Urinary fluoride
**Idiopathic**	
Paget disease	bsALP, XR, bone scan
**Genetic:** sclerosing bone dysplasias (includes, but not exclusive to):	
Osteopetrosis	Genetic testing
Pyknodysostosis	
Osteomesopyknosis	
Hyperostosis	
**Other**	
Hepatitis C-associated osteosclerosis (HCAO)	Hep C serology

A complete list of sclerosing bone disorders can be found in *Nosology of genetic skeletal disorders* (2023) by Unger et al [9].

Abbreviations: BMAT, bone marrow and trephine; bsALP, bone-specific alkaline phosphatase; BTM, bone turnover markers; CMP, calcium, magnesium, and phosphate; PTH, parathyroid hormone; TFT, thyroid function test; UEC, urea, electrolytes and creatinine; XR, x-ray.

SM is an uncommon neoplastic disease of mast cells, characterized by proliferation and accumulation in skin and other organs. It is diagnosed based upon minor and major criteria and is associated with a *c-KIT* gain-of-function mutation, which is found in >80% of cases [10, 11]. KIT-negative SM are very uncommon and associated with lower mast cell burden, variable tryptase levels and limited response to KIT-targeted therapies [11]. Mast cells in bone marrow may secrete mediators that promote osteoclastic or osteoblastic activity. It should be considered as a differential diagnosis in any individual presenting with atopic symptoms and unexplained osteoporosis/osteosclerosis. Management of indolent and smoldering mastocytosis is aimed at symptom control, with cytoreductive therapies generally reserved for advanced SM to mitigate organ damage and extend survival [12]. Our patient fulfilled at least 1 major criterion (multifocal dense infiltrates of mast cells with ≥15 mast cells in aggregates in bone marrow biopsies) and 1 minor criterion (persistently elevated serum tryptase >20 ng/mL, SI: >20 μg/L).

Once a diagnosis of SM is made, identification of “B and C” findings would highlight the degree of mast cell burden and organ involvement/dysfunction. This allows for classification of SM which may require targeted and systemic therapy [10]. These findings were absent in our patient.

Bone disease in mastocytosis either manifests as osteoporosis or sclerotic bone lesions. Osteoporosis is more common than osteosclerosis (50% of SM cases vs 5% to 6% of indolent SM cases) [4], although osteosclerosis is reported in up to 75% of cases with aggressive SM, which carries a poorer prognosis (median life expectancy of 41 months) [13]. The mechanism underlying mastocytosis-related bone disease remains poorly understood, but it is thought to be related to inflammatory mediators secreted by mast cells, resulting in an imbalance in osteoblastic and osteoclastic activity. Bone loss in mastocytosis has been associated with pro-inflammatory mediators (histamine, heparin, tumor necrosis factor [TNF] alpha, and interleukin (IL)-1, IL-6, IL-17) that lead to increased osteoclastic or reduced osteoblastic activity [4]. An immunosuppressive profile (lower interferon-gamma [IFN-γ] and IL-6) has been associated with mastocytosis-related osteosclerosis [4]. In contrast to osteoporosis cases, markedly elevated tryptase levels, higher levels of bone formation/turnover markers, and lower receptor activator of nuclear factor kappa-B ligand (RANKL) levels have been observed in diffuse bone disease compared to healthy controls [2]. In our patient, lack of end-organ involvement classified her as indolent SM. However, regular surveillance is highly recommended to monitor for transformation.

Although treatment of the underlying pathology remains key to reversing or preventing progression of osteosclerosis due to other causes, published literature is limited in the context of SM. Although one case report demonstrated resolution of osteosclerosis after allogenic stem cell hematopoietic stem cell transplant and subsequent cytoreductive therapy after one year [14], SM-related osteosclerosis is not a current indication for cytoreductive therapy. In contrast, studies have predominantly addressed osteoporosis associated with SM. Review of the literature has suggested improved BMD and suppression of bone turnover markers after use with bisphosphonates, in particular intravenous zoledronic acid [12], as well as denosumab [15] once alternative causes for osteoporosis have been considered [16]. However, a caveat to this includes the risk of developing an acute-phase reaction, which may be mitigated with pre-medications with simple analgesia and/or short-course corticosteroids [4]. Additionally, evidence for anabolic agents (eg, teriparatide) is limited and not recommended due to the theoretical risk of worsening mast cell proliferation and abnormal growth, especially if concerns for mast cell infiltration in bone [3].

In conclusion, we present a case of diffuse osteosclerosis secondary to mastocytosis and provided a comprehensive list of the differential diagnoses for sclerotic bone disease. The underlying mechanisms and clinical course associated with osteosclerosis remain poorly understood. This case contributes to the limited existing literature; however, further robust studies are required to clarify the pathogenesis and prognosis in SM-related osteosclerosis.

## Learning points

- Osteosclerosis is an uncommon finding but has significant clinical implications as it is frequently secondary to several serious conditions.
- Systemic mastocytosis-related bone disease is rare and should be considered in cases with atopic symptoms and unexplained osteoporosis/osteosclerosis.
- The mechanism underlying mastocytosis-related bone disease remains poorly understood but is thought to be related to inflammatory mediators secreted by mast cells, resulting in an imbalance in osteoblastic and osteoclastic activity.
- Treatment options for SM-related osteosclerosis are limited, while antiresorptive therapy has demonstrated efficacy in SM-associated osteoporosis.
- Further studies are required to better understand the underlying mechanism and clinical trajectory of SM-related osteosclerosis.

## Contributors

All authors made individual contributions to authorship. H.H.D. was involved in management of this patient and primary manuscript draft and submission. H.H.N., C.L., B.K., and V.G. were involved in the diagnosis and management of this patient and manuscript revision. All authors reviewed and approved the final draft.

## Data Availability

Data sharing is not applicable to this article as no datasets were generated or analyzed during the current study.

## Abbreviations

BMD: bone mineral density
CRP: C-reactive protein
CT: computed tomography
IL-: interleukin
SM: systemic mastocytosis
SPECT: single photon emission computed tomography
